# Exploring the mechanism of the Fructus Mume and Rhizoma Coptidis herb pair intervention in Ulcerative Colitis from the perspective of inflammation and immunity based on systemic pharmacology

**DOI:** 10.1186/s12906-022-03823-7

**Published:** 2023-01-16

**Authors:** Yatian Yang, Chengcheng Qian, Rui Wu, Rui Wang, Jinmei Ou, Shoujin Liu

**Affiliations:** 1 Anhui University of Chinese Medicine, Hefei, 230012 China; 2 Wuhu Institute of Technology, Wuhu, 241006 China; 3 Key Laboratory of Anhui Province for the New Technology of Chinese Medicine Decoction Pieces Manufacturing, Hefei, 230012 China; 4 Anhui Academy of Chinese Medicine Institute of Chinese Medicine Resources Protection and Development, Hefei, 230012 China

**Keywords:** Fructus Mume, Rhizoma Coptidis, Herb pair, Ulcerative colitis, Network pharmacology, Molecular docking, MAPK1, Inflammatory factor

## Abstract

**Purpose:**

Ulcerative Colitis (UC) is a chronic nonspecific inflammatory disease of the colon and rectum. Fructus Mume (FM) and Rhizoma Coptidis (RC) exert effects on inflammatory and immune diseases. We evaluated the hypothesis of the FM and RC (FM-RC) herb pair remedy in alleviating dextran sulfate sodium (DSS)-induced colitis, through network pharmacology-based analyses, molecular docking, and experimental validation.

**Methods:**

The Traditional Chinese medicine systematic pharmacology analysis platform(TCMSP) and Swiss database were used to predict potential targets of FM-RC and the GeneCards database was utilized to collect UC genes. Cytoscape software was used to construct and analyze the networks, and DAVID was utilized to perform enrichment analysis. AutoDock software was used to dock the core chemical components of the FM-RC herb pair with key UC targets. Animal experiments were performed to validate the prediction results and general conditions and body weight were observed. Pathological changes in colon tissue were observed by staining with hematoxylin and eosin. The levels of TNF-α, IL-8, IL-17, and IL-4 in serum and colon tissue were detected by ELISA.

**Results:**

Eighteen effective components of the herb couple were screened, and their potential therapeutic targets in the treatment of UC were acquired from 110 overlapped targets. GO and KEGG analyses revealed that these targets were highly correlated with protein autophosphorylation, plasma membrane, ATP binding, cancer pathways, the PI3K-AKt signaling pathway, and the Rap1 signaling pathway. Molecular docking established the core protein interactions with compounds having a docking energy < 0 kJ·mol^−1^, indicating the core active components had strong binding activities with the core targets. FM-RC herb pair relieved pathological indicators and reduced the concentration of TNF-α, IL-8, and IL-17 and increased IL-4 levels in the serum and colon tissues of UC rats.

**Conclusion:**

Collectively, FM-RC herb pair administration alleviated UC. These beneficial effects targeted MAPK1 signaling related to inflammation and immunity, which provided a basis for a better understanding of FM-RC in the treatment of UC.

## Introduction

Ulcerative Colitis (UC) is a nonspecific gastrointestinal inflammatory disease characterized by diarrhea, mucus, and blood in the stool [1]. Its pathogenesis is complex, and studies have shown that the disease is closely related to genetics, environmental factors, intestinal flora imbalance [2], immune disorders, and other factors [3]. At present, the incidence and prevalence of UC are increasing due to the irregularity of people’s diet and work and rest time. It is showing an increasing trend year by year, with an average of 1.2–20.3 and 7.6–24.5 cases per 100,000 people per year [4, 5]. The pathogenesis of UC is not clear, so far there is no targeted radical cure measures. Therefore, it is of certain significance to carry out research on the treatment of UC and related diseases.

Traditional Chinese herbal medicine has been widely used to treat UC for many years [6]. Wumei Pill is originated from Treatise on Cold Damage and Miscellaneous Diseases (200–210, AD), consisting of 10 herbs (as shown in Table 1) [7]. Wumei pill effectively relieve typical symptoms of UC, such as abdominal pain, diarrhea and loss of appetite [8–10]. Fructus Mume and Rhizoma Coptidis (FM-RC) as the main percent herb of Wumei Pill, their compatibility is commonly used in dysentery, diarrhea, intestinal adenoma and its carcinogenesis. Its mechanism of action may be related to the inhibition of inflammation and epithelial-mesenchymal transition [11, 12]. Previous studies have shown that the main components of FM are organic acids, flavonoids and fatty acids, and the main active ingredient is citric acid [13], the main components of RC include alkaloids, lignans, flavonoids, etc., and alkaloids are the main pharmacological components, berberine is the most abundant and representative compound [14]. Jiang et al. [15] showed that the compatibility of the herbal pair of FM-RC could alter the internal proportion of intestinal flora, promote the regulation of intestinal system balance, which played an important role in the treatment of UC. In addition, more and more studies have shown that FM not only has anti-tumor, antibacterial and anti-inflammatory effects, but also significantly improved weight loss and intestinal bleeding caused by UC, and inhibited expression of inflammatory factors such as TNF-α and IL-1β in the colon tissue of UC rats [16]. Li et al. [17] systematically evaluated the efficacy and safety of RC intervention in patients with UC to further guide its promotion and application. However, there is a lack of further research on the mechanism of FM-RC for the treatment of UC.

**Table 1:** The Composition of Wumei Pill

Herbal medicine	Chinese Name	Latin name	Quantity(g)
Fructus Mume	Wumei	*Prunus mume* Sieb.et Zuce	24
Rhizoma coptidis	Huanglian	*Coptis chinensis* Franch	24
Rhizoma Zingiberis	Ganjiang	*Zingiber officinale* Rosc	15
Rhizoma Radix Asari	Xixin	*Asarum heterotropoides* Fr. Schmidt	9
Radix Aconiti Lateralis Praeparata	Fuzi	*Typhonium giganteum* Engl	9
Ramulus Cinnamomi	Guizhi	*Cinnamomum cassia* Presl	9
Radix Ginseng	Renshen	*Panax ginseng* C. A. Meyer	9
Cortex Phellodender	Huangbai	*Phellodendron chinense* Schneid	9
Radix Angelicae Sinen sis	Danggui	*Angelica sinensis* (Oliv.) Diels	6
Pericarpium Zanthoxyli	Huajiao	*Zanthoxylum bungeanum* Maxim	6

Network pharmacology and molecular docking are new technologies based on systems biology and databased molecular correlation analysis in the exploration of new drugs and prediction of drug targets [18, 19]. Zhou et al. [20] combined the results of network pharmacology, molecular docking, and experimental verification indicated that FM promoted colorectal cell apoptosis and inhibited the development of colorectal cancer (CRC) mainly by inhibiting the expression of RelA. An et al. [21] with the help of a network pharmacology strategy, molecular docking and experimental validation, investigated that RC could be used to treat Type 2 Diabetes Mellitus (T2DM).To broaden the mechanism of FM-RC in the treatment of UC, using a system-integrated method to study the treatment of UC by FM-RC from the perspective of system level [22, 23].

Therefore, the aim of our study is to further consider the detailed mechanism of FM-RC herb pair in the treatment of UC systematically through network pharmacology, molecular docking and experimental validation in vivo, thereby providing new theoretical basis for Chinese medicine in the treatment of UC.

## Materials and methods

### Chemical composition database and active compound screening

We searched for the terms ‘Fructus Mume’ and ‘Rhizoma Coptidis’ in the Chinese Medicine Database and Analysis Platform (TCMSP) website. The bioavailability (OB) > 30% and drug similarity (DL) > 0.18, and main pharmacokinetic parameters, were used to screen the active components of FM-RC using the pharmacokinetic indexes of absorption, distribution, metabolism, and excretion (ADME) [24]. The OB value mainly reflects the proportion of a drug that can overcome a series of physiological barriers and be absorbed by the body and enter the blood circulation. The DL value mainly reflects the degree of similarity between the target molecule and the proven drug in the composition and eliminates inactive components in the chemical composition [25]. The combination of the two values is used to screen drug components. The target genes corresponding to the active ingredients of the drug were retrieved using the TCMSP (https://tcmsp-e.com/).

### Screening of possible targets for drug components

The chemical composition of the drugs was determined through the PubChem (https://pubchem.ncbi.nlm. nih.gov/) database to confirm the molecular structure, and was exported as a two-dimensional structure in an SDF file. The results were imported into the Swiss database (http://www.Swisstarget prediction.ch/) to predict the chemical targets of FM-RC. The UniProt database (https://www.uniprot.org) was used to limit data to ‘human species’, and the target names were normalized to obtain the official name and standard gene name of the compound targets.

### Predicting the possible UC targets

Data from target genes associated with UC were collected from GeneCards (https://www.genecards.org/) with the keywords ‘ulcerative colitis’ [26]. Genes from GeneCards were provided with scores, and genes with scores above the median degree were selected as UC-associated genes.

### Gathering compound-disease overlapped targets

The screened FM-RC targets and UC targets were imported into Bioinformatics (http://www.bioinformatics.com.cn/) [27], and the overlapped targets of compound-disease were obtained as the potential targets for further analysis.

### Construction of an active component-target network

A visual network was constructed using Cytoscape 3.7.2 software to reflect the complex relationship between active compounds and filtrated targets to reflect the relationship between included targets and active compounds [28]. The nodes represent the compounds and targets, while the edges indicate the interactions between the targets and components potentially included in the treatment of UC with FM-RC.

### Analysis of the protein–protein interaction network

The protein–protein interaction (PPI) network was derived based on the STRING database (https://string-db.org/). Species were set as ‘Homo sapiens’ and the ‘minimum required interaction score’ was set to 0.7 to ensure readability of the PPI network. This step hid nodes that were not related to each other, allowing the construction of the protein interaction diagram. The corresponding data files were imported into Cytoscape 3.7.1 software (http://www.cytoscape.org/), and the nodes were filtered with the median parameters of degree and betweenness centrality in Cyto NCA plug-in to obtain the core targets of UC treatment with FM-RC.

### Enrichment analysis for target proteins

By bioinformatics technology, with bioinformatics data analysis (www.bioinformatics.com.cn), Gene Ontology (GO) enrichment analysis, and Kyoto Encyclopedia of Genes and Genomes (KEGG) pathway analysis were performed to extract the biological functions of target proteins and cancer-related pathways. In addition, a histogram and bubble graph were drawn to visualize the data.

### Molecular docking

To validate the effectiveness of the screened compounds and targets, molecular docking was performed between the top 3 active compounds and the top 3 core targets. Compound structures in SDF format were downloaded from the PubChem database (https://pubchem.ncbi.nlm.nih.gov/) and converted to a mol 2 format file using Chem 3D software. The PDB format structures of SRC (PDB ID:2BDF), MAPK1(PDB ID:4NIF) and PIK3CA (PDB ID:4JPS) were downloaded from the RCSB Database (https://www.rcsb.org/). Pymol software was used to remove solvent molecules and ligands, and AutoDock Tools 1.5.6 software was used to hydrogenate, calculate charge, assign atomic types, and save the data in the PDBQT format.

### UPLC-QTOF-MS analysis

#### Preparation of the FM-RC herb pair extract

According to the ratio of 1:4, a total of 125 g of FM-RC was added to 8 times the amount of water, soaked in distilled water for 1 h cold and decocted for 1 h. The liquid was removed and 6 times the amount of water was added before boiling for 30 min. The liquid was poured out and combined twice. The liquid was filtered using a paper filter, and the residue was decompressed, and concentrated, freeze-dried, and weighed. The quantity of freeze-dried powder was 42.5 g and was stored until use.

#### UPLC-QTOF-MS conditions

The samples of 100 mg FM-RC extracts were weighed. After the addition of 1,000 μl extracting solution (methanol: water = 4:1, v/v, including internal standard concentration is 10 μg/ml), all samples were vortexed for 30 s, sonicated for 5 min in an ice-water bath, incubated at − 40 °C for 1 h, and centrifuged at 12,000 rpm at 4 °C for 15 min. A 500 μl of the supernatant was passed through a 0.22 μm filter membrane and then transferred to ultra-high-performance liquid chromatography tandem mass spectrometry (UHPLC-MS/MS) analysis.

LC–MS/MS analysis was performed on an UHPLC system (Vanquish, Thermo Fisher Scientific) with a Waters UPLC BEH C18 column (1.7 μm 2.1*100 mm). The sample injection volume was set at 5 μL. The flow rate was set at 0.5 mL/min. The mobile phase consisted of 0.1% formic acid in water (A) and 0.1% formic acid in acetonitrile (B). The multi-step linear elution gradient program was as follows: 0—11 min, 85—25% A; 11—12 min, 25—2% A; 12—14 min, 2—2% A; 14 – 14.1 min, 2—85% A; 14.1—15 min, 85—85% A; 15—16 min, 85—85% A. An Q Exactive Focus mass spectrometer coupled with an Xcalibur software was employed to obtain the MS and MS/MS data based on the IDA acquisition mode. During each acquisition cycle, the mass range was from 100 to 1500, and the top three of every cycle were screened and the corresponding MS/MS data were further acquired. Sheath gas flow rate: 45 Arb, Aux gas flow rate: 15 Arb, Capillary temperature: 350 ℃, Full ms resolution: 70,000, MS/MS resolution: 17,500, Collision energy: 15/30/45 in NCE mode, Spray Voltage: 4.0 kV (positive) or -4.0 kV (negative). Materials identification of peaks containing MSMS data was performed using the secondary mass spectrometry database (Shanghai BIOTREE biotech Co., Ltd.) and the corresponding cleavage law matching method.

### Experimental validation of FM-RC treatment in UC

#### Experimental animal model

SPF rats (male, 220 ± 20 g) were purchased from Huaxing Experimental Animal Farm, Huiji District, Zhengzhou City, animal license number SCXK (Yu) 2019–0002. All animals were housed in a room with constant temperature (25 ± 2 °C), relative humidity (50 ± 5%°C), half-day light–dark cycles, and free access to food and distilled water. Animal feeding and use were carried out in accordance with the relevant regulations of the Animal Ethics Committee of Anhui University of Chinese Medicine, ensuring that experiments were carried out in full compliance with the ‘Administrative Regulations on Laboratory Animals’ issued by the State Science and Technology Commission and the ‘Implementation Rules for the Management of Medical Laboratory Animals’ issued by the Ministry of Health.

#### Experimental drugs, reagents, and instruments

FM-RC, the origin of Sichuan Province, were identified by Associate Professor Ou Jinmei of Anhui University of Chinese Medicine as the near-ripe dried fruit of *Prunus mume* Sieb. et Zucc., and the rhizome of *Coptis chinensis* Franch. The following devices and reagents were used: mesalamine (Shanghai Sunway Pharmaceutical Company), the dextran sulfate sodium (DSS, MP Biomedicals, USA), Electronic balances (METTLER TOLEDO Instruments Co, Ltd), high-speed tissue grinder (Servicebio), desktop high-speed refrigerated centrifuge (Great Dragon), microplate detector (BioTeK), tumor necrosis factor-α (TNF-α), rat IL-4 ELISA kit, rat IL-8 ELISA kit, and rat IL-17 ELISA kit (Servicebio).

#### Establishment of the sodium dextran sulfate animal model and FM-RC treatment

After a 7-day adaption period, 32 SPF rats (Six-week-old male SD breeds) were randomly divided into four groups (*n* = 8 each): Control group (Con), DSS group (DSS), Mesalazine group (Mes), and FM-RC group (FM-RC). Rats in the control group received sterile water for 9 days and in other groups were given free access to a 3% DSS solution for 9 days to establish the UC model. On the second day of modeling, the Mes group received 0.42 g/kg Mes, intragastrically for 8 consecutive days, the FM-RC group received FM-RC1.26 g/kg, intragastrically for 8 consecutive days, while the normal group and the model group received sterile water. The herb pair accounts for a large proportion of FM, the dosage of the rats was determined according to the dosage of FM. The maximum dosage of FM recorded in the Chinese Pharmacopoeia is 12 g, based on the dose of the drug converted from the body surface area between humans and rats, it was determined that the dose of FM-RC to be administered to rats was 1.26 g/kg. The maximum dose of Mes for human is 4 g, and the dose for rats was calculated to be 0.042 g/kg in the same way. Body weight and disease activity index were monitored daily during administration, including weight loss, fecal hardness, and blood in the stool. DAI = (weight change score + stool trait score + blood in stool score) / 3. The DAI scoring criteria are shown in Table 2.

**Table 2:** Scoring criteria of disease activity index

Scoring	Percentage of body mass decrease	Stool viscosity	Stool bleeding
0	< 1%	Normal (formed, granular)	Normal
1	1% ~ 5%		
2	5% ~ 11%	Loose (mushy, not adhering to anus)	Occult blood
3	11% ~ 15%		
4	> 15%	Diarrhea (watery, adherent to anus)	Bloody stool

#### Specimen collection

After fasting for 24 h after the last administration, and 2% sodium pentobarbital was injected intraperitoneally for anesthesia. After blood collection, the rats were euthanized by cervical dislocation. Colon tissues were collected for indices detection and stored at -20 °C until further use.

#### Pathological observation of the rats colon

The colon tissue was fixed with 4% paraformaldehyde, decalcified, dehydrated, permeabilized, and embedded in paraffin for H&E staining. Histological changes were observed the microscope.

#### Serum detection of TNF-α, IL-4, IL-8, and IL-17

The blood samples of rats in each group were allowed to stand for 30 min, centrifuged (4 °C, 4000 rpm) for 10 min, and the upper serum fraction was obtained. TNF-α, IL-4, IL-8, and IL-17 in serum were determined using the Rat inflammation panel kit. The resulting data were analyzed by one-way ANOVA using GraphPad Prism 6.0. followed by Tukey’ s test for multiple comparisons.

#### Colon tissue detection of TNF-α, IL-4, IL-8 and IL-17

The colon tissues of rats in each group were added with 0.9% normal saline at a ratio of weight (mg): volume (μm) = 1:9, mechanically homogenized under an ice bath to prepare a 10% homogenate, and centrifuged (4 °C, 4000 rpm) for 10 min. The supernatant fraction was obtained. TNF-α, IL-4, IL-8, and IL-17 in colon tissue were measured using the Rat Inflammation Panel kit. The resulting data were analyzed by one-way ANOVA using GraphPad Prism 6.0. followed by Tukey’ s test for multiple comparisons.

## Results

### Potential active ingredients of FM-RC

The six component indexes of FM and 13 components of RC were retrieved from the TCMSP database. A total of 18 compounds were retrieved from the TCMSP database under the screening conditions of OB ≥ 30% and DL ≥ 0.18, of which quercetin was the common compound of the FM-RC (Table 3).

**Table 3:** Potential active components in FM-RC

Number	Molecular name	(OB)%	DL	Attribute
WM1	(2R)-5,7-dihydroxy-2-(4-hydroxyphenyl)chroman-4-one	42.36	0.21	FM
WM2	beta-sitosterol	36.91	0.75	FM
WM3	kaempferol	41.88	0.24	FM
WM4	stigmasterol	43.83	0.76	FM
WM5	methyl arachidonate	46.9	0.23	FM
WMHL	quercetin	46.43	0.28	FM,RC
HL1	berberine	36.86	0.78	RC
HL2	obacunone	43.29	0.77	RC
HL3	berberrubine	35.74	0.73	RC
HL4	epiberberine	43.09	0.78	RC
HL5	(R)-Canadine	55.37	0.77	RC
HL6	berlambine	36.68	0.82	RC
HL7	palmidin A	35.36	0.65	RC
HL8	palmatine	64.60	0.65	RC
HL9	coptisine	30.67	0.86	RC
HL10	worenine	45.83	0.87	RC
HL11	moupinamide	86.71	0.26	RC
HL12	corchoroside A_qt	104.95	0.78	RC

*Abbreviations:* *FM* Fructus Mume, *RC* Rhizoma Coptidis

### Targets related to the treatment of UC with FM-RC

**Fig. 1:**
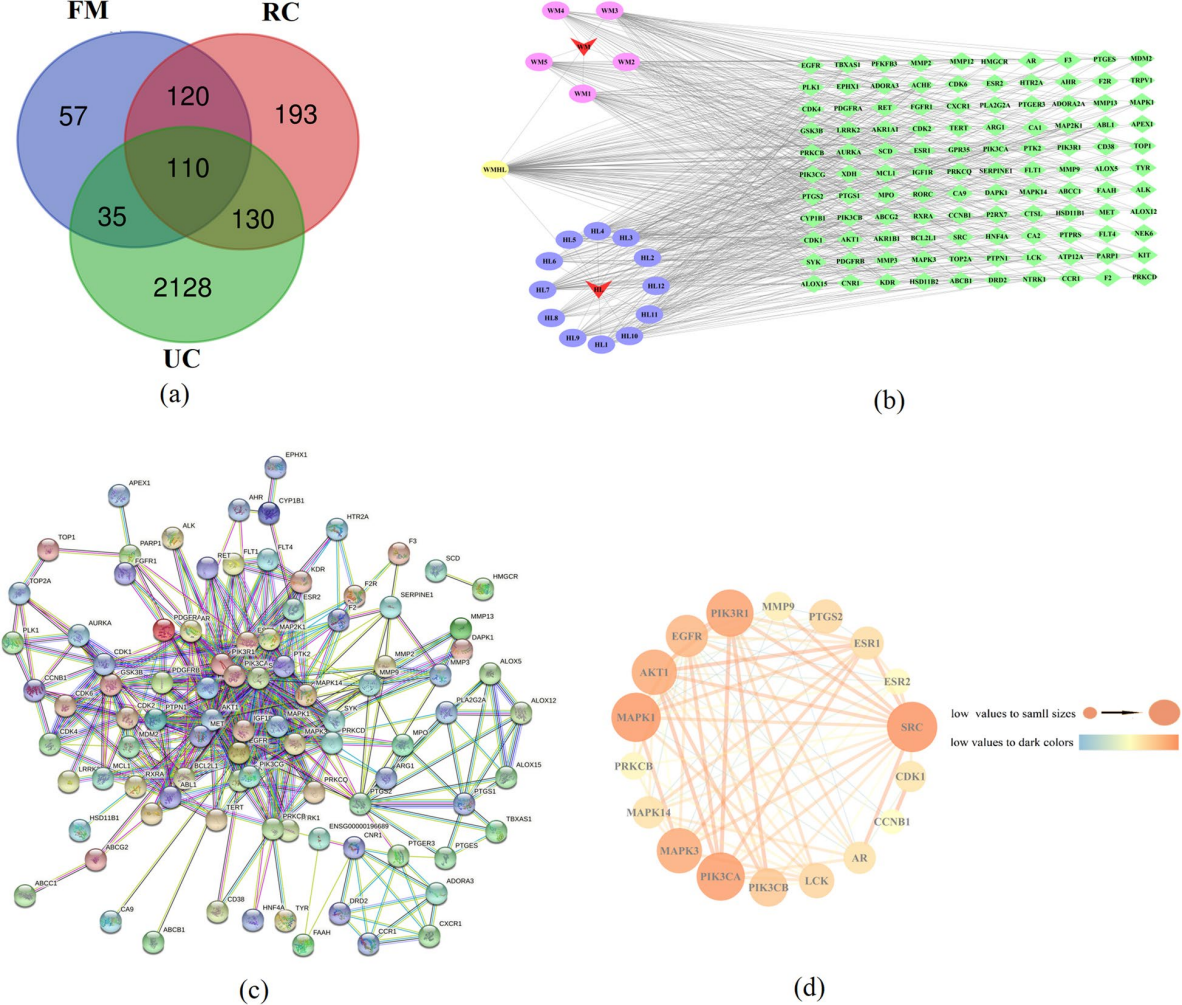
Network analysis results. **a** Venn diagram of potential targets for FM-RC and UC genes. **b** Medicinal material-active component-target-disease network (Note inverted triangle: drug; ellipse: compound; quadrilateral: protein target). **c** FM-RC-UC PPI network. **d** PPI network of core targets in the treatment of UC

The SDF structures of the active ingredients in Table 2 were queried through the PubChem database and imported into the Swiss database to identify the corresponding targets. A total of 1988 potential targets were collected, including 616 of FM and 1372 of RC. A total of 877 potential targets were obtained after gene normalization and deduplication of the collected targets through the UniProt database, including 322 for FM and 555 for RC. A total of 4806 targets related to UC were collected by searching the GeneCards database for disease-related keywords and 2403 disease targets were obtained after screening by the median of the relative score (RS). After the intersection of herb pair of compound targets and UC-related targets, 110 overlapping targets were obtained, which was shown by a Venn diagram (Fig. 1a).

### Construction and analysis of FM-RC targets-UC differential gene expression network

Using Cytoscape network visualization software, a drug-target interaction map was constructed, as shown in Fig. 1(b). There are 130 nodes in the network (including 2 for FM-RC, 18 compounds, and 110 gene targets). The degree value of a node represents the number of lines connected to the node in the network, and the higher the degree value, the more likely the compound will work. Topological analysis was carried out on the characteristics of the network relationship and the results are shown in Table 4. The top 5 nodes in the degree value were WMHL, WM3, HL7, WM1, and WM5, that is, quercetin, kaempferol, palmidin A, (2R)-5, 7-dihydroxy-2-(4-hydroxyphenyl)chroman-4-one and methyl arachidonate were key compounds in the treatment of UC. In Fig. 1b, it appeared that the active components of FM-RC acted synergistically on multiple targets, such as beta-sitosterol in FM and berberine in RC cooperating on the PTPN1, BCHE, ACHE, HSD11B1, SIGMAR1, CDC25B, TBXAS1, SRC and MAPK14 targets; kaempferol and stigmasterol in FM, obacunone in RC and quercetin were shared by the two drugs and were synergistically active on the expression of ACHE, CYP19A1, SRC, KDR and BACE1, suggesting that the combined use of the two drugs may be more effective than the single drug preliminarily.

**Table 4:** Rank table of degree values of compounds in FM-RC

Number	Compounds	Betweenness	Degree
WMHL	quercetin	0.17689236	110
WM3	kaempferol	0.15900131	55
HL7	Palmidin A	0.11956144	45
WM1	(2R)-5,7-dihydroxy-2-(4-hydroxyphenyl)chroman-4-one	0.10293098	41
WM5	methyl arachidonate	0.09429742	36
WM4	stigmasterol	0.06046575	31
WM2	beta-sitosterol	0.06018728	31
HL3	berberrubine	0.03941812	30
HL12	Corchoroside A_qt	0.06176487	28
HL9	coptisine	0.03997748	28
HL2	Obacunone	0.0542441	27
HL11	Moupinamide	0.03001212	26
HL4	epiberberine	0.03470086	26
HL6	Berlambine	0.02793021	25
HL5	(R)-Canadine	0.02427034	24
HL8	palmatine	0.02709629	23
HL10	Worenine	0.01865624	22
HL1	berberine	0.01587965	22

### Construction of the PPI network

One hundred ten targets were uploaded to the STRING database for analysis. We selected protein targets with a medium confidence score of 0.700, which were plotted as an interaction network. The PPI network was established through the STRING database. As shown in Fig. 1(c), a topological analysis was performed in Cytoscape 3.7.1 software, and the average of two parameters, degree and betweenness, was used as the reference standard for screening. A total of 18 key targets were obtained, which as the key targets of FM-RC in the treatment of UC, and the network construction analysis was carried out based on them. As shown in Fig. 1(d), the size and color of the node represented the size of the degree value. The larger the node, the darker the color, and the larger the corresponding degree value. Depending on the degree value, the top 8 protein nodes were selected, namely SRC, MAPK1, PIK3CA, PIK3R1, AKT1, MAPK3, EGFR and PIK3CB.

### GO enrichment analysis and KEGG pathway enrichment

**Fig. 2:**
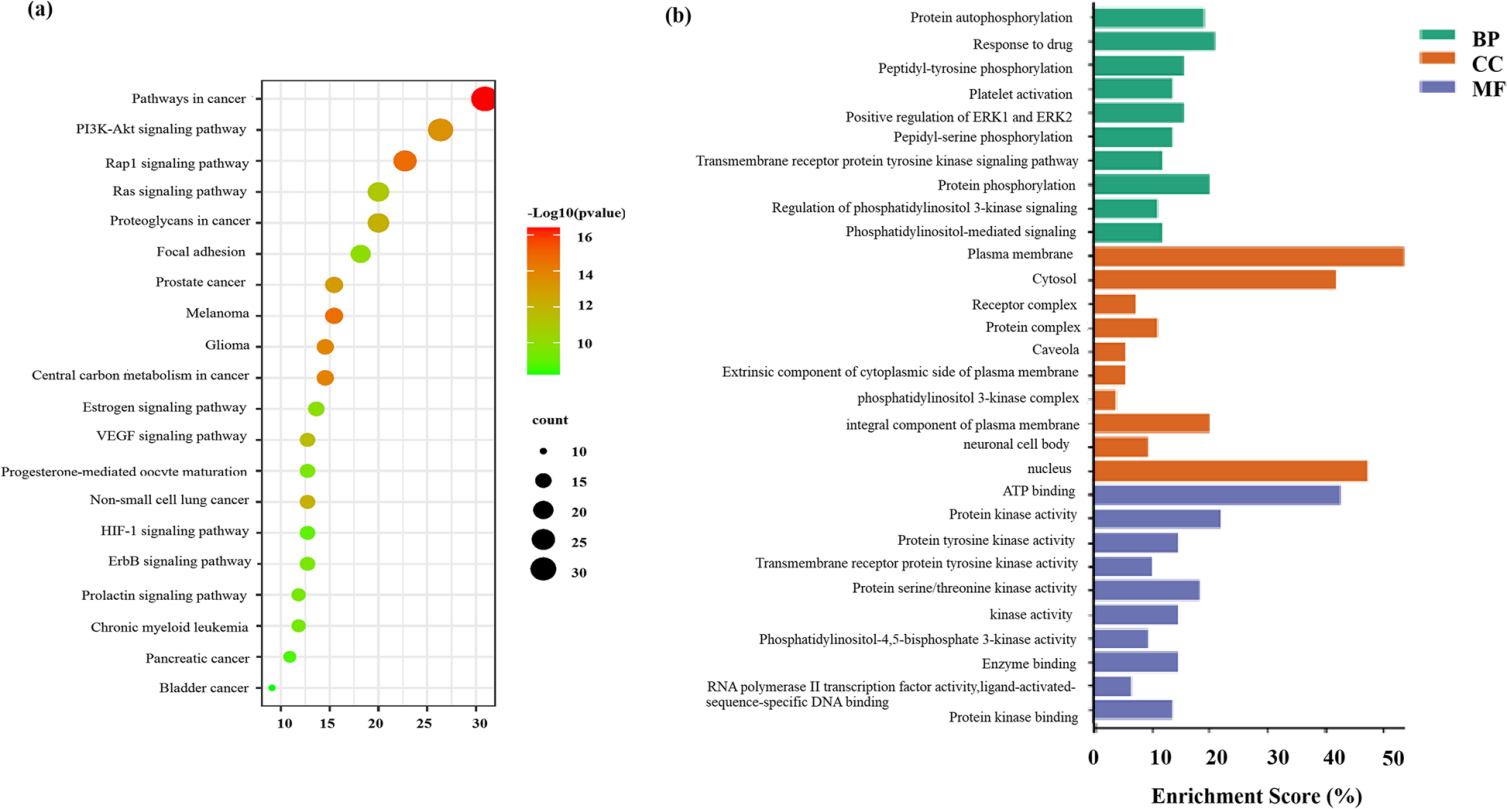
Target biological function and target-pathway analysis. **a** Molecular Function (MF), Cell Components (CC) and Biological Processes(BP) of FM-RC in the treatment of UC. **b** Signal pathway of FM-RC in the treatment of UC

Using the DAVID database, GO enrichment analysis was performed on 110 potential targets and set *P* < 0.05. A total of 398 biological processes or pathways were obtained, of which 77 were related to molecular function (MF), 56 were related to the cellular component (CC), and 265 were related to the biological process (BP). According to the increasing order of the *P*-value, the top 10 proteins were selected and a bar graph was drawn, as shown in Fig. 2(a). In the BP, protein autophosphorylation, response to drug, and peptidyl-tyrosine phosphorylation were ranked the top pathways; in CC, plasma membrane, cytosol and receptor complex ranked high; Among the MF, ATP binding, protein kinase activity, and protein tyrosine kinase activity ranked the top.

To further reveal the potential mechanism of FM-RC on the effect of UC, we conducted a KEGG pathway enrichment analysis on 110 targets and selected 94 pathways based on the threshold of *P* < 0.05. The first 20 pathways were selected and incorporated into a bubble chart. The larger the bubble, the more genes enriched in the GO entry (Fig. 2b). The results revealed the signaling pathways activated by FM-RC, and included pathways in cancer, the PI3K-AKt signaling pathway, the Rap1 signaling pathway, the Ras signaling pathway, and proteoglycans in cancer.

### Molecular docking

**Fig. 3:**
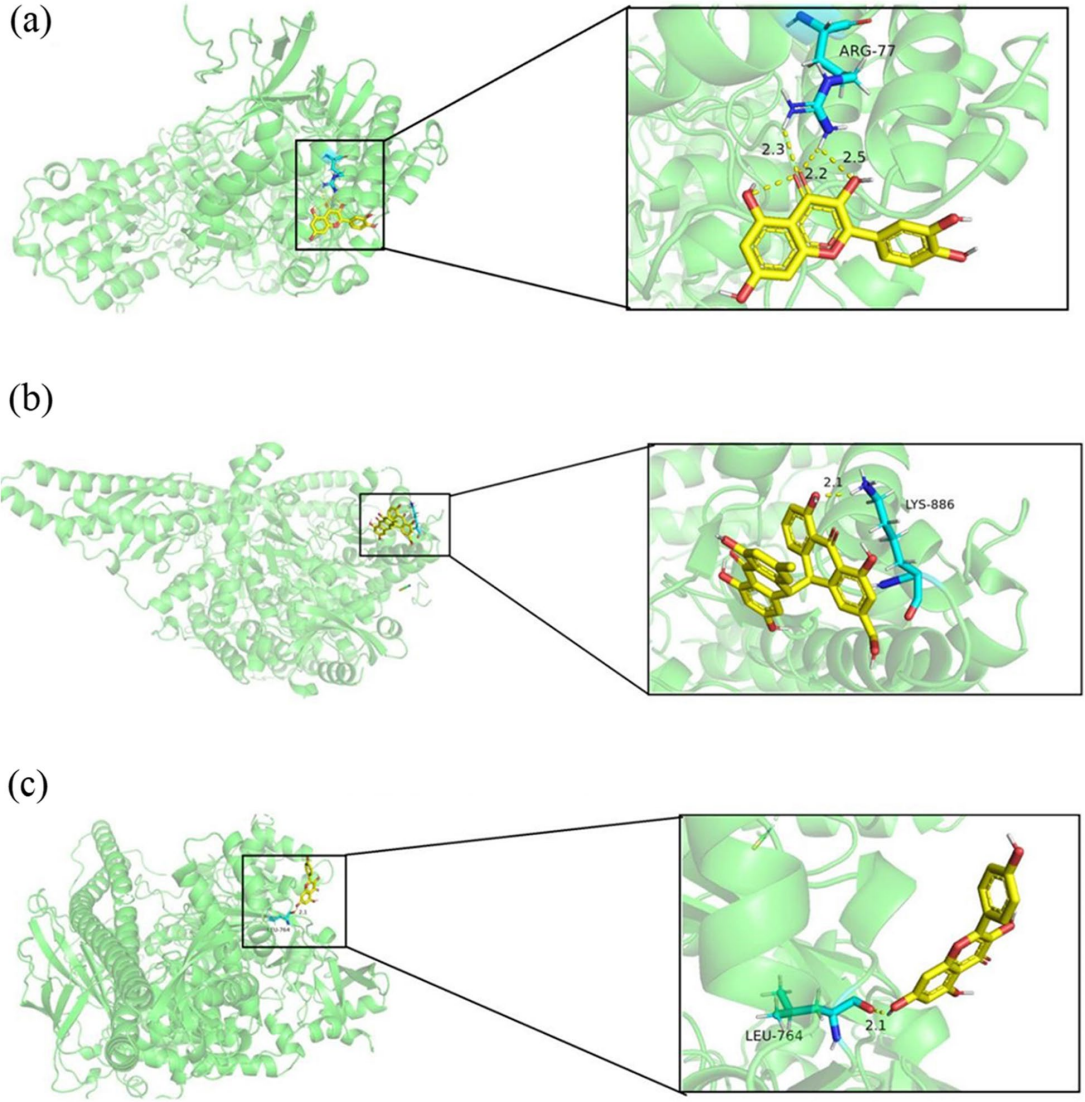
Docking diagram of core active components and core targets of FM-RC in the treatment of UC. (**a**) The ligand-receptor interaction screening of quercetin and MAPK1. (**b**) The ligand-receptor interaction screening of Palmidin A and PIK3CA. (**c**) The ligand-receptor interaction screening of kaempferol and PIK3CA

Molecular docking was applied to analyze the binding of the top 3 key target proteins (SRC [PDB ID: 2BDF], MAPK1 [PDB ID: 4NIF], PIK3CA [PDB ID: 4JPS]) and the top 3 key active ingredients (quercetin, kaempferol, and palmidin A), as shown in Table 5. The docking fraction were summarized in Table 3. The lower the score, the higher the interaction intensity. Quercetin and MAPK1 proteins had the smallest docking energy, followed by palmidin A and PIK3CA proteins, and finally kaempferol and PIK3CA proteins, which were visualized by pymol in Fig. 3.

**Table 5:** Molecular docking binding energy

Ligand component	Receptor protein	Binding energy( kJ·mol^−1^)
quercetin	SRC	-0.92
quercetin	MAPK1	-2.6
quercetin	PIK3CA	-2.1
kaempferol	SRC	-1.74
kaempferol	MAPK1	-2.11
kaempferol	PIK3CA	-2.35
Palmidin A	SRC	-2.24
Palmidin A	MAPK1	-0.43
Palmidin A	PIK3CA	-2.43

### Chemical profiling of FM-RC extracts

**Fig. 4:**
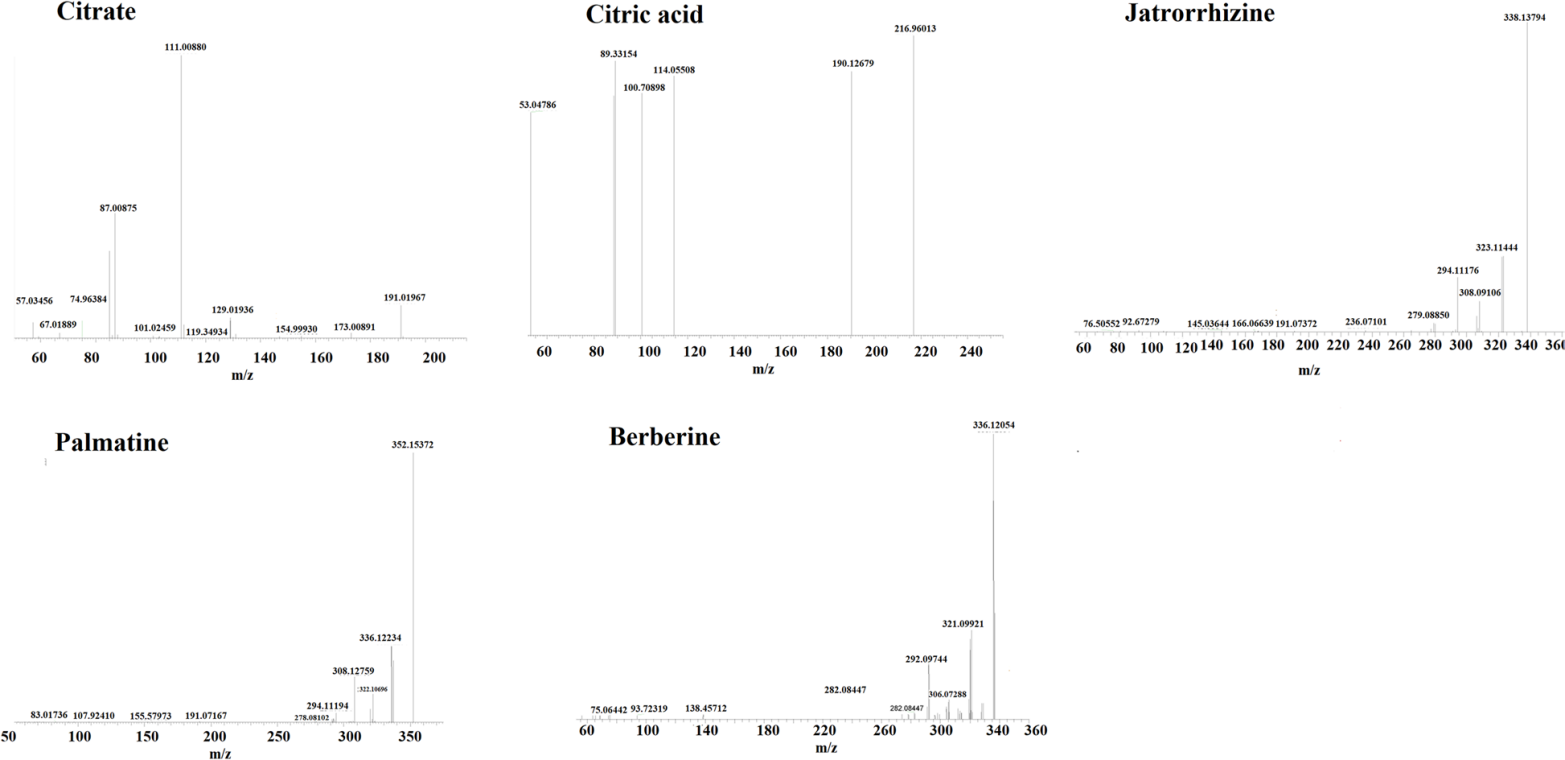
Secondary mass spectra of Citrate, citric acid, Jatrorrhizine, Palmatine, Berberine in FM-RC

To control the quality of the herbal extract, the FM-RC extracts samples were analyzed using UHPLC-MS/MS. The total positive and negative ion chromatograms of FM-RC demonstrated the chemical composition of all compounds, Berberine, Palmatine, Citric acid, Epiberberine and Quercetin were found in FM-RC extracts, and semi-quantified by relative peak area (Table 6). The secondary mass spectra of the compounds were shown in Fig. 4. Significantly, The decoction process of the FM-RC herb pair may undergo salt formation reactions due to the acidic and the alkaline components, and the Citrate is the main ingredient in the FM-RC extracts. Additionally, It has been reported that Citric acid as a material basis for the anti-colitis activity of FM [28]. Niu et al. [29] suggested that Jatrorrhizine plays a protective role in DSS-induced colitis by regulating the intestinal barrier function and inhibiting the TLR4/MyD88/NF-κB signaling pathway. Palmatine protected mice against DSS-induced colitis by facilitating PINK1/Parkin-driven mitophagy and thus inactivating NLRP3 inflammasomes in macrophage [30]. Berberine could ameliorate UC by maintaining the epithelial barrier via up-regulating the expression of tight junction proteins [31].

**Table 6:** Mass spectral data of five active ingredients in FM-RC

Compounds	t_R_/S	Formula	MS^2^(m/z)	ms2Adduct	semi-quantified
Citrate	32.3554	C6H8O7	191.0195636	[M-H]-	30.0576%
Citric acid	32.7498	C6H8O7	230.98985	[M + K] +	1.6328%
Jatrorrhizine	105.356	C20H20NO4 +	338.1375832	[M +]	22.8929%
Palmatine	154.236	C21H22NO4	352.1535889	[M] +	13.0933%
Berberine	199.595	C20H18NO4 +	336.1223259	[M +]	14.3281%

### FM-RC alleviated DSS-induced UC

**Fig. 5:**
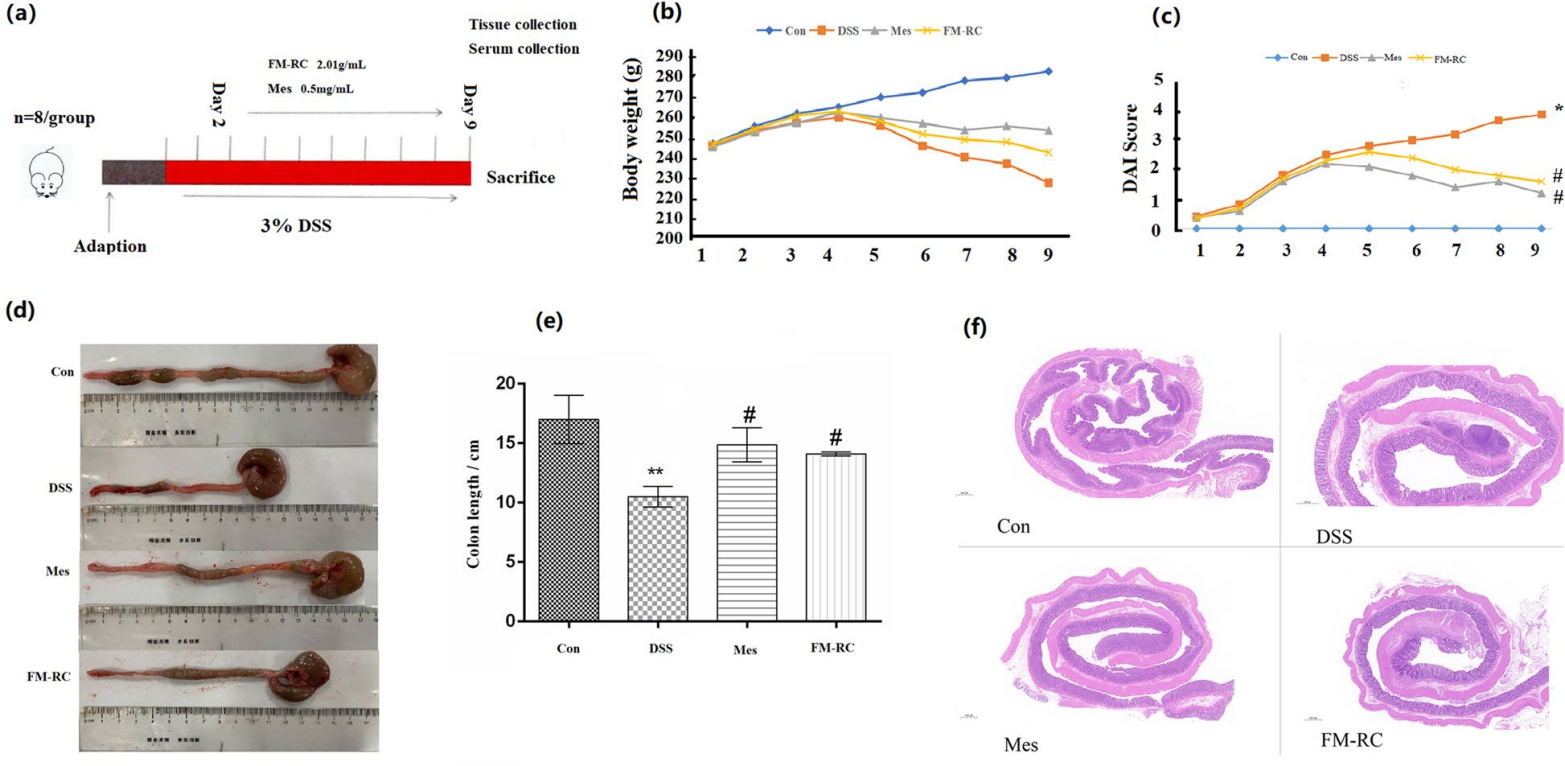
FM-RC alleviated DSS-induced colitis. **a** Scheme of the animal experimental design. Rats were randomly assigned to four groups (*n* = 8). Rats were continuously administered sterile water for 9 days. Colitis was induced by drinking 3% DSS from days 0 to 9, followed by 2 days of FM-RC receiving FM-RC. On day 9, the rats were sacrificed. **b** The body weight change of four groups. **c** The DAI scores calculated with time. **d** Representative colons of four groups and **e** the colon length after DSS treatment (right). **f** Representative H&E staining images of colon tissues sections from rats per group. Data are shown as the mean ± SEM. * *P* < 0.05 versus the control group, ** *P* < 0.01versus the control group. # *P* < 0.05 versus the DSS group. # #*P* < 0.05 versus the DSS group (the same applies below)

To further explore the effects of FM-RC herb pair extracts on UC in vivo, we established the DSS–induced rats UC model and treated rats with FM-RC simultaneously, as shown in Fig. 5a. DSS-treated rats exhibited more severe colitis, as indicated by the appearance of decreased weight, increased DAI score, and shortened colon length compared to control rats (Fig. 5b–e). According to HE staining (Fig. 5f), there was no abnormal histological changes in the control group. In the DSS group, the colonic epithelial tissue was ulcerated with inflammatory cell infiltration, and the entire intestinal wall was thinned, as well as the continuous structure of the crypt was destroyed. Compared to the DSS group, treatment with FM-RC extract restored part of the damaged intestinal structure and improved the pathological changes described above.

### Effects of FM-RC on DSS-induced secretion of inflammatory cytokines in serum

**Fig. 6:**
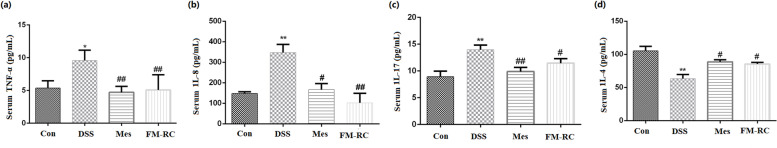
Effects of FM-RC on DSS-induced secretion of inflammatory cytokines TNF-α(**a**), IL-8(**b**), IL-17(**c**) and IL-4(**d**) in the serum

In the present study, the ELISA assay was used to assess inflammatory cytokine secretions in DSS-treated rats. Elevated protein levels of TNF-α, IL-8, and IL-17 in serum were observed in the DSS group compared to the control group. FM-RC significantly suppressed up-regulation of these inflammatory cytokines (Fig. 6a–c). Furthermore, DSS treatment reduced IL-4 protein levels in serum compared to the control group. However, the effects were significantly increased by FM-RC administration (Fig. 6d).

### Effects of FM-RC on DSS-induced secretion of inflammatory cytokines in colon tissues

**Fig. 7:**
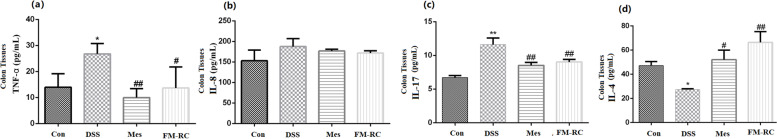
Effects of FM-RC on DSS-induced secretion of inflammatory cytokines TNF-α(**a**), IL-8(**b**), IL-17(**c**) and IL-4(**d**) in the colon tissues

Consistent with the inflammatory cytokines in the serum results, the levels of inflammatory cytokines in the colon tissues showed that TNF-α, IL-8 and IL-17 in the model group exhibited higher levels compared to those of the control group (Fig. 7a-c). However, the increase was inhibited by treatment with FM-RC. Furthermore, DSS-treated rats also showed increased IL-8 protein levels, but no differences were observed between the DSS group and the FM-RC group (Fig. 7b). DSS treatment reduced IL-4 protein levels in colon tissues compared to the control group, however, the effects increased significantly increased by FM-RC administration (Fig. 7d). These results indicated that FM-RC administration eliminated TNF-α and IL-17 pro-inflammatory levels in DSS challenge and enhanced IL-4 synthesis. Moreover, restoration of IL-8 levels was observed with exposure to FM-RC, although limited in extent, and may contribute to the protective function.

## Discussion

In the present study, a protective effect of FM-RC on the intestine was investigated in a model of DSS-induced colitis. Our data showed that FM-RC administration attenuated DSS-induced body weight loss, diarrhea, and colon shortening in rats. Further analysis demonstrated that DSS administration led to an increase in the concentrations of TNF-α, IL-8, and IL-17 concentrations in serum and colon tissues, as well as reduced protein levels of IL-4 in serum and colon tissues of rats. Notably, DSS-induced colonic damage, as indicated by histological alterations, was greatly attenuated by FM-RC administration. These beneficial effects of FM-RC were associated with quercetin and MAPK1 signaling.

From the TCMSP database, Swiss, and other databases, a total of 18 potential active ingredients of FM-RC, 110 common targets in the herb pair and UC were identified. Subsequently, we further screened essential genes and core-dependent pathways through the PPI network. The Database for Annotation, Visualization and Integrated Discovery (DAVID, https://david.ncifcrf.gov/) provides systematic, comprehensive biological annotation information for large-scale genes or proteins, and provides the most significantly enriched biological annotations. The DAVID web server was adopted to conduct GO enrichment analysis for the candidate target protein obtained after network merging. Subsequently, KEGG pathway enrichment analysis was conducted to explore biological pathways where relevant proteins were covered. A *P* value ≤ 0.05 was considered signifcant, and enriched GO terms were identifed by the hypergeometric test. A bubble plot of bioprocess and pathways were drawn by bioinformatics data analysis (www.bioinformatics.com.cn). Ultimately, the three bioactive compounds (quercetin, kaempferol, and palmidin A) and genes (SRC, MAPK1, and PIK3CA) with the highest differential expression within the constraints and the apoptosis-associated pathway were identified. Quercetin is a flavonoid molecule [32]. Ye et al. [33] studied the mechanism of quercetin in the treatment of Rheumatoid Arthritis (RA) and confirmed that quercetin exerted various biological activities such as inhibition of inflammatory cytokines, antioxidants, and immune regulation.Kaempferol is a polyphenol antioxidant [34]. Rajendran et al. [35] studied the mechanism of kaempferol in the prevention of CRC, indicating that kaempferol can reduce the expression of the CRC inhibitor DACT2 gene by regulating DNMT and HDAC proteins, and can also inhibit the migration of metastatic tumors. SRC is a non-receptor tyrosine protein kinase. The increased activity of SRC kinase not only reduces the adhesion between tumor cells but also increases the permeability of endothelial cells, thereby promoting the metastasis of tumor cells [36, 37]. Therefore, regulating the activity of SRC kinase plays a key role in the occurrence and development of UC. MAPK1, or mitogen-activated protein kinase 1, is involved in various pathophysiological processes such as gene transcription, apoptosis, cell growth, and immune response. When external factors cause activation of MAPK protein, NF-kB is activated by MAPK phosphorylation with specific substrates and translocated into the nucleus, where transcriptases initiate gene expression programs that regulate the production of inflammatory factors such as TNF-α, IL-8, and IL-17, exacerbating the inflammatory response [38–40]. PIK3CA regulates cell proliferation, differentiation, apoptosis, and other functions by activating the PI3K-AKT-mTOR pathway. When this gene was mutated, the PI3K-AKT-mTOR pathway could be abnormally activated, leading to the occurrence of colorectal cancer [41]. We then performed molecular fitting scoring and molecular docking for the top three genes and bioactive components. The results indicated that quercetin and MAPK1 were bioactive compounds and genes, respectively, with the best affinity and reasonable degree in FM-RC for the treatment of UC.

Accumulating evidence has shown that overactivation of the inflammatory response contributes to the initiation and development of IBD or DSS-induced colitis. TNF-α, IL-8, and IL-17 are considered key pro-inflammatory cytokines that lead to the development of UC [42, 43]. TNF-α is a monokine produced by monocytes and macrophages, which can promote the occurrence of an inflammatory response [44]. IL-8 is an inflammatory cytokine promoted by TNF-α and participates in the intestinal inflammatory response by chemotaxis of basophils and T cells and promote neutrophil adhesion and activation [45]. IL-17 is mainly secreted by T helper 17 cells, which not only induce endothelial cells and epithelial cells to analyze inflammatory factors such as IL-8 but also increase intestinal mucosa permeability and recruit neutrophils to induce inflammation of the intestinal mucosa of UC [46]. Conversely, IL-4 is an important anti-inflammatory cytokine with multiple functions [47], which can effectively inhibit the synthesis and antigen presentation of pro-inflammatory cytokines, thus reducing the inflammatory response, leading to the appearance of UC.

In this study, we combined the results of network pharmacology, molecular docking, and experimental in animal models at three levels. Among the bioactive ingredients associated with UC in FM-RC, quercetin and MAPK showed the highest degree of molecular docking. We also verified the effects of inflammatory factors associated with the MAPK pathway on the animal model of UC.

However, this study also presented some limitations. First, the data on various drugs, genes, and proteins were not comprehensive, and appropriate computing software has not been developed. Second, the bioactive ingredients of FM-RC should be verified individually by experiments. Finally, it is necessary to explore the direct regulation of bioactive ingredients on MAPK1 in future studies.

## Conclusion

Through network pharmacology and molecular docking, the study preliminarily revealed the main active components, targets, and signaling pathways of FM-RC therapy UC, Meanwhile, we performed an animal experiment to confirm that the herb pair of FM-RC interferes with UC through the MAPK1 signaling pathways associated with inflammation and immunity. Specifically, FM-RC herb pair regulates the immune function by affecting the expression levels of TNF-α, IL-8, IL-17, and IL-4. These results provide a scientifific basis for the prevention and treatment of UC.

## Data Availability

All data generated or analyzed in this study are included in this article.

## Abbreviations

UC: Ulcerative Colitis
FM: Fructus Mume
RC: Rhizoma Coptidis
DSS: Dextran sulfate sodium
TCMSP: Traditional Chinese Medicine Systems Pharmacology Database
DL: Drug Likeness
OB: Oral Bioavailability
PPI: Protein–protein interaction
KEGG: Kyoto Encyclopedia of Genes and Genomes
GO: Gene Ontology
CRC: Colorectal cancer
T2DM: Type 2 Diabetes Mellitu
